# SNP variation in the promoter of the *PRKAG3* gene and association with meat quality traits in pig

**DOI:** 10.1186/1471-2156-13-66

**Published:** 2012-07-25

**Authors:** Marion T Ryan, Ruth M Hamill, Aisling M O’Halloran, Grace C Davey, Jean McBryan, Anne M Mullen, Chris McGee, Marina Gispert, Olwen I Southwood, Torres Sweeney

**Affiliations:** 1 School of Veterinary Medicine, University College Dublin Belfield, Dublin 4, Ireland; 2 Functional Genomics & Glycomics Group, Martin Ryan Institute, National University of Ireland Galway, Galway, Ireland; 3 Teagasc, Ashtown Food Research Centre, Ashtown, Dublin 15, Ireland; 4 IRTA, 17121 Monells, Girona, Spain; 5 Genus PLC/PIC, Alpha Building, London Road, Nantwich, CW5 7JW, UK

**Keywords:** Promoter activity, Gene expression, Transcription factor binding site, Gene expression, Single nucleotide polymorphisms

## Abstract

**Background:**

The *PRKAG3* gene encodes the γ3 subunit of adenosine monophosphate activated protein kinase (*AMPK*), a protein that plays a key role in energy metabolism in skeletal muscle. Non-synonymous single nucleotide polymorphisms (SNPs) in this gene such as *I199V* are associated with important pork quality traits. The objective of this study was to investigate the relationship between gene expression of the *PRKAG3* gene, SNP variation in the *PRKAG3* promoter and meat quality phenotypes in pork.

**Results:**

*PRKAG3* gene expression was found to correlate with a number of traits relating to glycolytic potential (GP) and intramuscular fat (IMF) in three phenotypically diverse F1 crosses comprising of 31 Large White, 23 Duroc and 32 Pietrain sire breeds. The majority of associations were observed in the Large White cross. There was a significant association between genotype at the g.-311A>G locus and *PRKAG3* gene expression in the Large White cross. In the same population, ten novel SNPs were identified within a 1.3 kb region spanning the promoter and from this three major haplotypes were inferred. Two tagging SNPs (g.-995A>G and g.-311A>G) characterised the haplotypes within the promoter region being studied. These two SNPs were subsequently genotyped in larger populations consisting of Large White (n = 98), Duroc (n = 99) and Pietrain (n = 98) purebreds. Four major haplotypes including promoter SNP’s g.-995A>G and g.-311A>G and *I199V* were inferred. In the Large White breed, HAP1 was associated with IMF% in the *M. longissmus thoracis et lumborum* (LTL) and driploss%. HAP2 was associated with IMFL% GP-influenced traits pH at 24 hr in LTL (pHULT), pH at 45 min in LTL (pH_45_LT) and pH at 45 min in the *M. semimembranosus* muscle (pH_45_SM). HAP3 was associated with driploss%, pHULT pH_45_LT and b* Minolta. In the Duroc breed, associations were observed between HAP1 and driploss% and pHUSM. No associations were observed with the remaining haplotypes (HAP2, HAP3 and HAP4) in the Duroc breed. The Pietrain breed was monomorphic in the promoter region. The *I199V* locus was associated with several GP-influenced traits across all three breeds and IMF% in the Large White and Pietrain breed. No significant difference in promoter function was observed for the three main promoter haplotypes when tested *in vitro.*

**Conclusion:**

Gene expression levels of the porcine *PRKAG3* are associated with meat quality phenotypes relating to glycolytic potential and IMF% in the Large White breed, while SNP variation in the promoter region of the gene is associated with *PRKAG3* gene expression and meat quality phenotypes.

## Background

Adenosine monophosphate activated protein kinase (*AMPK*) is a heterodimeric serine/threonine protein kinase. This enzyme is a metabolic master regulator of several intracellular pathways, including cellular uptake of glucose, glycogen synthesis and β-oxidation of fatty acids, controlling metabolism through transcription and direct effects on metabolic enzymes [1-3]. *AMPK* is composed of a catalytic α-subunit and two regulatory non-catalytic β- and γ- subunits. A specific isoform of the regulatory γ subunit of *AMPK* (*AMPK* γ3) has a role in the metabolic plasticity of fast-glycolytic muscle [4]. The *AMPK* γ3 isoform is encoded by the highly conserved *PRKAG3* gene and is primarily expressed in white (fast-twitch, type IIb) skeletal muscle fibers [5,6]. Gain of function mutations in the *PRKAG3* gene have been correlated with increased glycogen content in skeletal muscle in pig [7,8], mice [9] and humans [10]. The metabolic consequences of these mutations extend beyond glycogen metabolism however, and can also influence other characteristics of the muscle, including mitochondrial biogenesis [11], fatty acid uptake [10,12] oxidative capacity [12] and differential responses to exercise [8].

Five non-synonymous substitutions (*T30N, G52S, L53P, I199V* and *R200Q*) have been reported in the porcine *PRKAG3* gene [7,13,14]. The effects of *I199V* and *R200Q* are the most widely studied. Both mutations are located in a highly conserved region of the cystathionine β- synthase domain [13] which is believed to act as a sensor of cellular energy status [15]. The *R200Q* polymorphism has been linked to a 70% increase in glycogen content in the skeletal muscle of Hampshire pigs. The resultant alteration in GP affects meat quality traits including water holding capacity (drip loss%) and pH, as well as processing yields [13], but the SNP has largely been eliminated from breeding populations [16]. The adjacent polymorphism *I199V*[7] has also been associated with glycolytic traits including water holding capacity [17] and pH [18]. The minor I allele is widely reported to have positive effect on pH and water holding capacity in diverse pig breed populations [7,17-19]. The physiological functions of *PRKAG3* contribute to a wider range of pork quality characteristics including colour [18,20,21], carcass composition [22,23], seasoning losses [24] and IMF% [25,26].

Preliminary data from our group has suggested that the *PRKAG3* gene is differentially expressed in LTL muscle of pigs displaying extremes in the distribution of drip loss% values in a Large White population [27]. Hence, our first objective was to test for an association between *PRKAG3* gene expression and pigmeat quality phenotypes, with a focus on GP-influenced traits and IMF%. This was performed in 3 sire breeds (Large White, Duroc and Pietrain) that are divergent in their muscle and meat quality characteristics [28]. Alterations in gene expression can be caused by genetic variation in the regulatory regions of the gene [29-31]. Hence, the second objective was to identify novel genetic variation in the *PRKAG3* gene promoter region. The final objective was to examine if the identified allelic variants differed in promoter function by: a) testing for association between novel SNPs/haplotypes and gene expression and pork quality traits, and b) investigating how haplotype variation affects putative transcription factor binding sites *in silico* and promoter function *in vitro.*

## Results

### Relationship between *PRKAG3* expression and pork quality phenotypes

A number of correlations were identified between *PRKAG3* [GenBank; NM_214077.1] expression levels in the LTL muscle and meat quality phenotypes which are known to be influenced by glycolytic potential in the muscle (termed GP-influenced traits) in the F1 crossbred pig populations (Table 1). The Large White population displayed the greatest number of significant correlations, with a smaller number of correlations observed in the Duroc cross breed, while none were observed in the Pietrain cross breed. In the Large White cross breed a number of traits were positively correlated with *PRKAG3* gene expression including water holding capacity measures driploss% and cookloss%. ECULT and colour Minolta L* were also positively correlated with *PRKAG3* gene expression while pHULT was negatively correlated. A smaller number of correlations with GP- influenced traits were evident in the Duroc cross breed including pHULT (negative) and Minolta L* on Day 7 (positive). IMF % was also positively correlated with *PRKAG3* gene expression in the Large White cross breed.

**Table 1:** **Pearson correlation (r**^**2**^**) between *PRKAG3 *gene expression and phenotypic measurements in 3 crossbred pig population: Large White, Duroc and Pietrain-sired F1 female offspring with a common Large White x Landrace background**

**Trait category**	**Trait**	**Large white X**	**Duroc X**	**Pietrain X**
		**n = 30**	**n = 23**	**n = 31**
Glycolytic Potential	Driploss%	0.562***	−0.167	0.278
	Cookloss% D1	0.503***	0.241	0.002
	Cookloss% D7	0.354*	−0.015	0.074
	pHULT	−0.504***	−0.44*	−0.312
	ECULT	0.383*	−0.396	0.241
	Colour L* LT D1	0.540***	0.363	0.232
	Colour L* LT D3	0.436**	0.210	0.149
	Colour L* LT D7	0.362*	0.42*	0.199
Intra Muscular Fat	IMF%	0.438**	−0.246	0.130

Significance denoted by * = <0.05, ** = <0.01, *** = <0.005.

Associations significant at the Bonferroni-adjusted alpha are underlined. Bonferroni-adjusted alpha levels were as follows for single SNP association analysis: Large White =0.015, Duroc =0.014, Pietrain =0.013.

### SNP Discovery and identification of tagging SNPs

**Figure 1:**
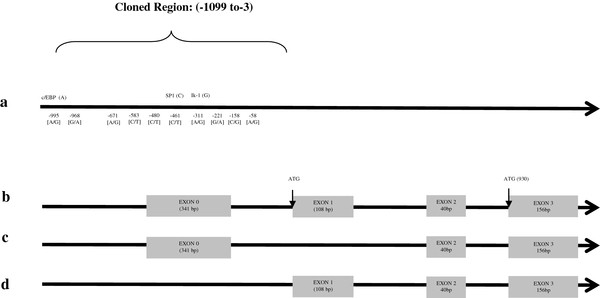
**Overview of SNPs in promoter region of *PRKAG3 *gene and outline of alternative transcripts.**

**Figure 2:**
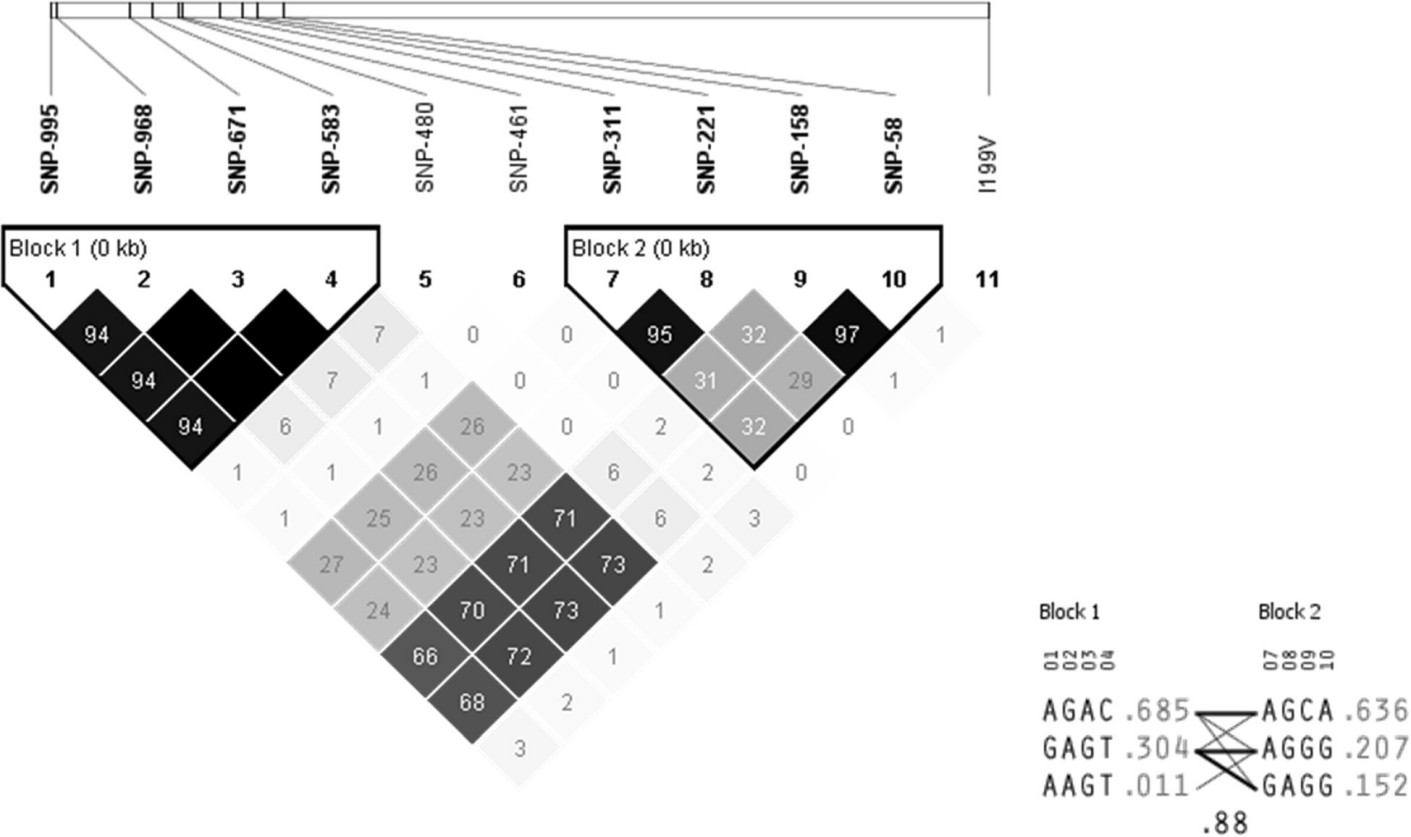
**Output from Haploview including linkage disequilibrium (LD) plots indicating r**^2 ^**values for SNPs in the regulatory region and inferred haplotypes for entire crossbred populations; Large White X (n = 30), Duroc X (n = 23) Pietrain X (n = 32).**

Sequencing of the 1322 bp upstream region revealed 10 novel SNPs (Figure 1) in the crossbred population. Minor Allele Frequencies (MAF) in the F1 population were variable for each cross breed and are outlined in Table 2. Two haplotype blocks were identified in the promoter region in these F1 populations; comprising 4 SNPS in each (Figure 2). Linkage disequilibrium was not significant between any of the SNPs in the promoter region and the non-synonymous SNP *I199V* in the coding region in the crossbred populations (Figure 2).

**Table 2:** Novel SNPS in the promoter region, and * *I199V *non synonymous SNP (previously published), including positions relative to transcription start site, base pair change and minor allele frequency in 3 crossbred pig populations: Large White, Duroc and Pietrain-sired F1 female offspring with a common Large White x Landrace background

**SNP 1>2**	**NCBI SNP submission #**	**SNP position relative to TSS**	**Allele frequency**		
			**Large white X**	**Duroc X**	**Pietrain X**
			**n = 30**	**n = 23**	**n = 32**
g.-995A>G	472333124	−995	0.45 (G)	0.28 (G)	0.23 (G)
g.-968G>A	472333125	−968	0.48 (A)	0.28 (A)	0.23 (A)
g.-671A>G	472333126	−671	0.48 (G)	0.28 (G)	0.23 (G)
g.-583C>T	472333127	−583	0.48 (T)	0.28 (T)	0.23 (T)
g.-480C>T	472333128	−480	0.05 (T)	0.05 (T)	0.00 (T)
g.-461C>T	472333129	−461	0.00 (T)	0.17 (T)	0.00 (T)
g.-311A>G	472333130	−311	0.36 (G)	0.07 (G)	0.11 (G)
g.-221G>A	472333131	−221	0.37 (A)	0.07 (A)	0.11 (A)
g.-158C>G	472333132	−158	0.42 (C)	0.30 (G)	0.27 (G)
g.-58A>G	472333133	−58	0.43 (A)	0.30 (G)	0.27 (G)
*I199V*		2774	0.34	0.17	0.42

The three major haplotypes were identified in the promoter region; AGACAGCA, GAGTGAGG and GAGTAGGG which accounted for 80% of the total variation in the Large White, 90% in the Pietrain and 93% in Duroc (Table 3). Genotypes at the g.-995A>G and g.-311A>G loci permitted resolution of these three major promoter haplotypes in all three F1 populations.

**Table 3:** Promoter haplotype frequencies for pure bred populations: Large White (n = 98), Duroc (n = 99) and Pietrain (n = 98)

**Promoter haplotype**	**Haplotype frequency**		
	**Large white**	**Duroc**	**Pietrain**
	**(n = 98)**	**(n = 99)**	**(n = 98)**
**A**GAC**A**GCA	0.750	0.495	0.995
**G**AGT**G**AGG	0.240	0.296	0.005
**G**AGT**A**GGG	0.010	0.209	0.000

Genotypes were tested for association with *PRKAG3* gene expression. A significant association (*P* =0.03) was observed between genotype at the g.-311A>G position and *PRKAG3* gene expression in the Large White breed. AA genotypes displayed the lowest gene expression (Mean = 0.31, ± 0.03), heterozygotes intermediate (Mean = 0.43, ± 0.04) and GG the highest (Mean = 0.51, ± 0.06). No association between the g.-995A>G SNP and gene expression was observed in any cross breed.

### Genotyping of tagging SNP’s in larger purebred populations

**Figure 3:**
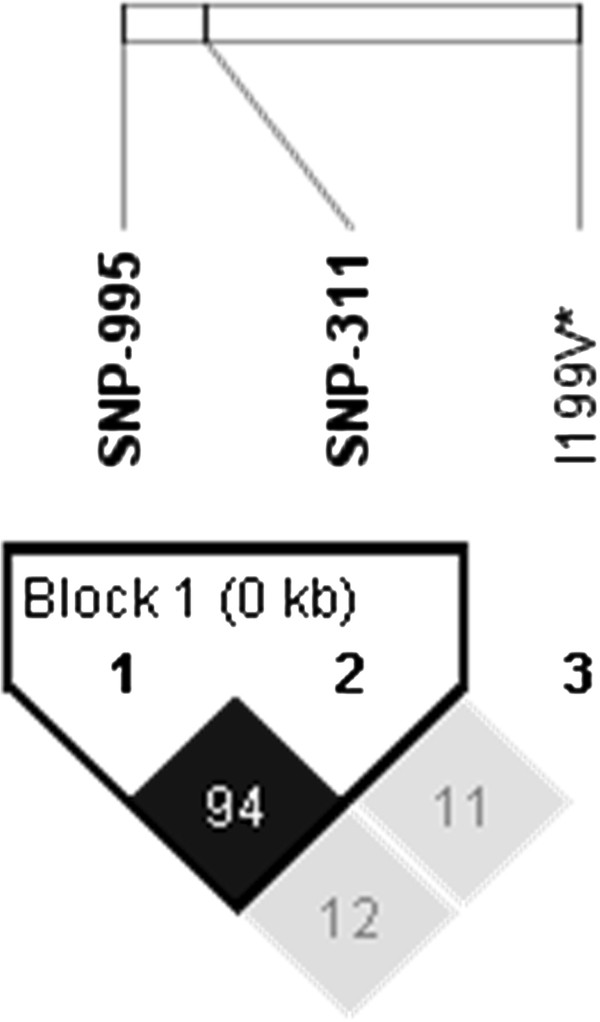
**Output from Haploview including linkage disequilibrium (LD) plots indicating r**^2 ^** values for SNPs in the regulatory region and inferred haplotypes for purebred Large White population (n = 98).**

**Figure 4:**
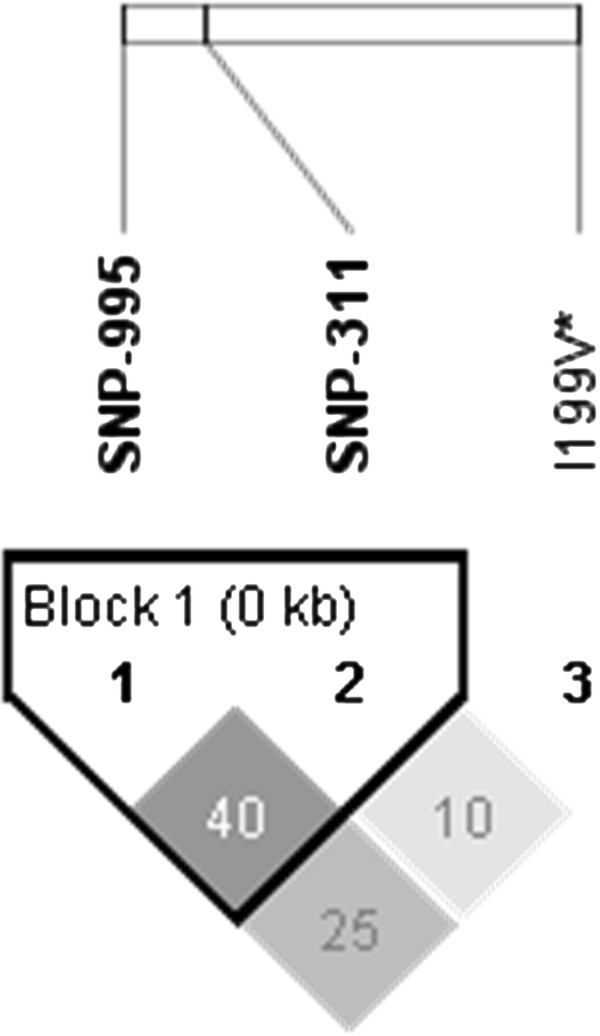
**Output from Haploview including linkage disequilibrium (LD) plots indicating r**^2 ^**values for SNPs in the regulatory region and inferred haplotypes for purebred Duroc population (n = 99).**

Genotypes were obtained for 2 SNPs in the promoter region (g.-995A>G and g.-311A>G) and the previously reported non-synonymous SNP *I199V* in three purebred populations. SNPs g.-995A>G and g.-311A>G in the promoter region were polymorphic in the Large White (Minor Allele Frequency (MAF): 0.25 and 0.24 respectively) and Duroc (MAF: 0.49 and 0.29 respectively) breeds. Pietrain animals were almost completely monomorphic for both these SNPs (MAF, 0.051 and 0.053, respectively). All breeds were polymorphic at the *I199V* locus. Linkage disequilibria plots showed no significant linkage disequilibrium between the two tagging SNPs in the promoter region and the *I199V* SNP in either the crossbred (Figure 2) or purebred (Figures 3 &4) populations. Genotype frequencies were in agreement with Hardy Weinberg equilibrium.

### *I199V* and associations with GP traits and IMF%

SNP *I199V* was associated with a number of GP-influenced traits in all three pure breeds (Table 4). The association with driploss% was observed in all breeds, where the II genotype had the lowest driploss% and the VV genotype had the highest driploss%. The effect was additive in all breeds, a 47%, 54% and 31% decrease in driploss% was observed from VV to II, in Large White, Duroc and Pietrain breeds respectively. In the Duroc breed, there was a small but significant additive association with ultimate pH at 24 hr in the *M. semimembranosus* muscle (pHUSM), with the II genotype having the highest and VV the lowest pH values. In the Pietrain breed, there were associations with electrical conductivity in the SM muscle (ECUSM), again the effect was additive with a 22% reduction in conductivity from VV to II. Minolta a* was associated with *I199V* in this breed, the heterozygote AG genotype had a lower colour score (~26%) compared to both homozygotes.

**Table 4:** **Estimated least squares means for meat quality traits in relation to *I199V *locus in 3 purebred Large White and Duroc and Pietrain animals**

**Trait**	**Large white**				**Duroc**				**Pietrain**			
	**p-value**	**II**	**IV**	**VV**	**p-value**	**II**	**IV**	**VV**	**p-value**	**II**	**IV**	**VV**
**Glycolytic traits**		**n = 6**	**n = 41**	**n = 51**		**n = 4**	**n = 30**	**n = 64**		**n = 30**	**n = 45**	**n = 21**
Driploss%	**0.016**	1.66 (0.49)	2.66 (0.18)	3.09 (0.15)	**0.039**	1.21 (0.54)	2.26 (0.20)	2.58 (0.13)	**0.016**	2.46 (0.2)	2.90 (0.19)	3.57 (0.30)
pHULT	0.656	5.67 (0.05)	5.63 (0.01)	5.63 (0.02)	0.164	5.67 (0.07)	5.66 (0.02)	5.63 (0.01)	0.459	5.66 (0.02)	5.65 (0.02)	5.61 (0.03)
pHUSM	0.591	5.57 (0.04)	5.55 (0.02)	5.53 (0.01)	**0.015**	5.65 (0.04)	5.54 (0.02)	5.53 (0.01)	0.165	5.58 (0.02)	5.55 (0.06)	5.52 (0.02)
pH_45_LT	0.216	6.68 (0.03)	6.56 (0.03)	6.60 (0.10)	0.491	6.44 (0.11)	6.56 (0.04)	6.57 (0.03)	**0.053**	6.52 (0.02)	6.60 (0.02)	6.51 (0.04)
pH_45_SM	0.420	6.60 (0.03)	6.50 (0.03)	6.55 (0.09)	0.717	5.67 (0.05)	5.67 (0.02)	5.63 (0.01)	0.333	6.41 (0.04)	6.45 (0.04)	6.34 (0.06)
ECULT	0.605	2.81 (0.32)	3.10 (0.12)	3.15 (0.10)	0.096	2.60 (0.44)	3.47 (0.16)	3.13 (0.10)	0.072	3.28 (0.12)	3.12 (0.10)	3.60 (0.16)
ECUSM	0.212	4.75 (0.53)	3.78 (0.20)	4.05 (0.17)	0.745	4.19 (0.41)	3.89 (0.15)	4.00 (0.10)	**0.019**	4.08 (0.25)	4.23 (0.20)	5.23 (0.32)
**Colour**												
L*	0.842	47.10 (1.49)	46.20 (0.57)	46.54 (0.48)	0.300	44.25 (1.49)	45.72 (0.53)	46.38 (0.35)	**0.0004**	45.90 (0.65)	47.40 (0.53)	46.00 (0.85)
b *	0.978	3.57 (0.68)	3.70 (0.26)	3.64 (0.22)	0.123	2.94 (0.69)	3.07 (0.24)	3.69 (0.16)	0.167	3.81 (0.19)	2.80 (0.25)	3.68 (0.25)
**Fatty Acids**												
IMF% LT	**0.035**	1.48 (0.20)	1.04 (0.08)	0.93 (0.07)	0.086	1.87 (0.31)	2.03 (0.11)	1.71 (0.07)	**0.002**	1.43 (0.07)	1.20 (0.06)	1.01 (0.09)
IMF% SM	0.316	1.13 (0.06)	1.25 (0.07)	1.36 (0.19)	0.858	2.14 (0.38)	2.22 (0.14)	2.12 (0.09)	0.356	1.56 (0.08)	1.46 (0.07)	1.37 (0.10)

P-values significantly different at the nominal level are in bold. Associations significant at the Bonferroni-adjusted alpha are underlined.

Bonferroni-adjusted alpha levels were as follows for single SNP association analysis: Large White =0.0083, Duroc =0.0083, Pietrain =0.0089.

*IMF* Intramuscular Fat, *LT M. Longissimus thoracis et lumborum* muscle, *SM M. semimembranosus* muscle, *n* number of genotypes.

 SNP *I199V* was associated with IMF% in the LTL muscle in both Large White and Pietrain breeds. The II genotype had the highest IMF% and VV the lowest in both breeds. The effect was additive, with a 37% and 29.4% decrease in IMF% from the II to VV genotype, in Large White and Pietrain, respectively.

### SNP g.-995A>G

The major allele (A) was fixed in the Pietrain breed. Association analysis of g.-995A>G and meat quality traits in Large White and Duroc breeds are presented in Table 5. In the Large White breed this SNP was associated with a range of GP-influenced traits. AA animals had a 34.5% decrease in driploss% relative to the alternative homozygote (GG) genotype, with the AG genotype driploss% values intermediate. Samples with alternative AA/GG genotypes thus differed by more than 1.2% in total driploss%. Similarly AA genotype animals had higher pHULT, pH_45_LT, lower ECULT, and a tendency for lower Minolta L* and b* values. No significant associations were observed in the Duroc population.

**Table 5:** Estimated least squares means for meat quality traits in relation to g.-995A>G and g.-311A>G SNP genotypes in purebred Large White and Duroc animals

**Trait**	**Large white**								**Duroc**							
	**g.-995A>G Promoter region**				**g.-311A>G Promoter region**				**g.-995A>G Promoter region**				**g.-311A > G Promoter region**			
	**p-value**	**AA**	**AG**	**GG**	**p-value**	**AA**	**AG**	**GG**	**p-value**	**AA**	**AG**	**GG**	**p-value**	**AA**	**AG**	**GG**
**Glycolytic Traits**		**n = 55**	**n = 37**	**n = 6**		**n = 56**	**n = 37**	**n = 5**		**n = 20**	**n = 54**	**n = 22**		**n = 45**	**n = 47**	**n = 5**
Driploss%	**0.002**	2.41 (0.19)	3.22 (0.18)	3.68 (0.44)	**0.009**	2.46 (0.16)	3.22 (0.18)	3.46 (0.51)	0.204	2.08 (0.27)	2.46 (0.15)	2.77 (0.23)	0.181	2.38 (0.16)	2.37 (0.15)	3.29 (0.47)
pHULT	**0.0003**	5.67 (0.01)	5.59 (0.02)	5.52 (0.04)	**0.0002**	5.67 (0.01)	5.60 (0.02)	5.43 (0.05)	0.345	5.67 (0.02)	5.63 (0.01)	5.63 (0.02)	0.172	5.65 (0.01)	5.64 (0.01)	5.56 (0.04)
pHUSM	0.637	5.55 (0.01)	5.55 (0.02)	5.51 (0.04)	0.639	5.55 (0.01)	5.54 (0.02)	5.50 (0.05)	0.684	5.55 (0.02)	5.53 (0.01)	5.54 (0.02)	0.710	5.53 (0.04)	5.53 (0.01)	5.51 (0.04)
pH_45_LT	**0.038**	6.60 (0.03)	6.51 (0.03)	6.51 (0.08)	**0.056**	6.67 (0.03)	6.56 (0.03)	6.60 (0.10)	0.767	6.58 (0.05)	6.55 (0.03)	6.59 (0.05)	0.827	6.55 (0.03)	6.57 (0.03)	6.51 (0.09)
pH_45_SM	0.139	6.6 (0.03)	6.62 (0.03)	6.51 (0.03)	0.118	6.60 (0.03)	6.50 (0.03)	6.60 (0.09)	0.684	6.57 (0.05)	6.47 (0.03)	6.51 (0.05)	0.811	6.51 (0.03)	6.51 (0.03)	6.45 (0.09)
ECULT	**0.029**	2.93 (0.10)	3.34 (0.12)	3.47 (0.30)	0.109	2.97 (0.10)	3.27 (0.11)	3.55 (0.34)	0.130	3.20 (0.20)	3.38 (0.11)	2.93 (0.19)	0.890	3.23 (0.12)	3.25 (0.12)	3.06 (0.38)
ECUSM	0.069	3.73 (0.17)	4.17 (0.20)	4.87 (0.50)	0.200	3.70 (0.17)	4.20 (0.20)	4.10 (0.57)	0.310	3.90 (0.17)	3.83 (0.10)	4.13 (0.16)	0.454	4.04 (0.10)	3.85 (0.10)	3.97 (0.32)
**Colour**																
L *	**0.064**	45.62 (0.47)	47.44 (0.55)	47.28 (1.40)	0.077	45.65 (0.47)	47.31 (0.55)	47.88 (1.55)	0.785	45.83 (0.68)	46.20 (0.39)	46.51 (0.64)	0.997	46.09 (0.41)	46.13 (0.41)	46.16 (1.27)
b *	**0.052**	3.27 (0.22)	4.02 (0.25)	4.52 (0.64)	**0.023**	3.26 (0.21)	3.99 (0.25)	5.05 (0.70)	0.706	3.30 (0.32)	3.49 (0.18)	3.69 (0.30)	0.502	3.42 (0.19)	3.44 (0.19)	4.15 (0.59)
**Fatty Acids**																
IMF% LT	0.467	0.95 (0.07)	1.04 (0.08)	1.19 (0.20)	0.343	0.94 (0.07)	1.05 (0.08)	1.25 (0.22)	0.918	1.86 (0.15)	1.79 (0.08)	1.82 (0.14)	0.538	1.84 (0.09)	1.83 (0.09)	1.53 (0.27)
IMF% SM	0.536	1.15 (0.06)	1.24 (0.07)	1.27 (0.17)	0.334	1.13 (0.06)	1.25 (0.07)	1.36 (0.19)	0.689	2.23 (0.17)	2.09 (0.10)	2.21 (0.16)	0.869	2.14 (0.10)	2.18 (0.10)	2.02 (0.32)

*P*-values significantly different at the nominal level are in bold. Associations significant at the Bonferroni-adjusted alpha are underlined.

Bonferroni-adjusted alpha levels were as follows for single SNP association analysis: Large White =0.0083, Duroc =0.0083.

*IMF* Intramuscular Fat, LT *M. Longissimus thoracis et lumborum* muscle, SM *M. semimembranosus muscle, n* number of genotypes.

### SNPg.-311A>G

There was almost complete linkage observed between g.-995A>G and g.-311A>G in the purebred Large White breed population (Figure 3) and to a lesser degree in the purebred Duroc population (Figure 4). All significant associations with g.-311A>G were observed in the Large White breed and mirror those reported for g.-995A>G.

### Association analysis between *PRKAG3* haplotypes for promoter SNPS and *I199V* and pork quality

Four major haplotypes comprising SNP g.-995A>G, SNPg.-311A>G and *I199V* were inferred by the Arlequin software [32]. Associations for all haplotypes and estimated least square means for meat traits per haplotype copy are outlined for the Large White and Duroc Breeds in Tables 6 and 7 respectively. HAP2 was the most frequent haplotype in the Large White breed: (Freq: 0.47). All haplotypes were relatively evenly represented in the Duroc breed HAP1 (Freq: 0.19), HAP 2 (Freq: 0.28), HAP 3 (Freq: 0.28) and HAP4 (Freq: 0.21). As the promoter SNPs were fixed in the Pietrain breed, haplotype association analysis was not performed for this breed.

**Table 6:** **Estimated least square means for significant meat quality traits in relation to copy number of the most abundant porcine PRKAG3 gene haplotypes as inferred by Arlequin for the promoter SNPs g.-995A>G and SNPg.-311A>G and non-synonymous SNP ***I199V ***locus in the Large White breed (n = 98)**

**HAP ID**	**Traits**	***P*- value**	**Estimated trait mean per haplotype copy**		
			**0**	**1**	**2**
HAP1 (AAI) (Freq: 0.27)	IMFL%	**0.04**	0.93 (0.06)	1.04 (0.08)	1.48 (0.20)
	IMFS %	0.32	1.18 (0.06)	1.17 (0.07)	1.46 (0.18)
	Driploss%	**0.02**	3.09 (0.15)	2.66 (0.18)	1.66 (0.51)
	pHULT	0.66	5.63 (0.02)	5.63 (0.02)	5.67 (0.05)
	pHUSM	0.59	5.54 (0.01)	5.55 (0.02)	5.58 (0.04)
	pH_45_LT	0.22	6.67 (0.03)	6.58 (0.04)	6.59 (0.09)
	pH_45_SM	0.42	6.59 (0.03)	6.53 (0.03)	6.51 (0.09)
	ECULT	0.60	3.15 (0.10)	3.14 (0.12)	2.81 (0.32)
	ECUSM	0.21	4.06 (0.17)	3.78 (0.20)	4.75 (0.53)
	L* Minolta	0.84	46.5 (0.48)	46.2 (0.57)	47.1 (1.49)
	b* Minolta	0.98	3.64 (0.22)	3.70 (0.26)	3.57 (0.69)
HAP2 (AAV) (Freq: 0.47)	IMFL%	**0.01**	1.16 (0.09)	1.03 (0.07)	0.76 (0.11)
	IMFS %	0.25	1.27 (0.08)	1.21 (0.06)	1.07 (0.09)
	Driploss%	0.59	2.84 (0.22)	2.95 (0.18)	2.64 (0.24)
	pHULT	**0.03**	5.60 (0.02)	5.63 (0.02)	5.68 (0.02)
	pHUSM	0.95	5.55 (0.02)	5.55 (0.01)	5.54 (0.02)
	pH_45_LT	**0.003**	6.55 (0.04)	6.61 (0.03)	6.75 (0.04)
	pH_45_SM	**0.04**	6.49 (0.04)	6.56 (0.03)	6.64 (0.04)
	ECULT	0.23	3.22 (0.14)	3.19 (0.11)	2.89 (0.15)
	ECUSM	0.13	4.29 (0.23)	4.01 (0.18)	3.57 (0.25)
	L* Minolta	0.12	47.1 (0.65)	46.6 (0.51)	45.3 (0.70)
	b* Minolta	0.12	4.02 (0.29)	3.72 (0.23)	3.10 (0.32)
HAP3 (GGV) (Freq: 0.24)	IMFL%	0.34	0.94 (0.07)	1.05 (0.08)	1.26 (0.22)
	IMFS %	0.33	1.13 (0.06)	1.25 (0.07)	1.36 (0.19)
	Driploss%	**0.01**	2.46 (0.16)	3.23 (0.18)	3.46 (0.51)
	pHULT	**0.0002**	5.67 (0.01)	5.60 (0.02)	5.49 (0.05)
	pHUSM	0.64	5.55 (0.01)	5.54 (0.02)	5.50 (0.04)
	pH_45_LT	**0.06**	6.68 (0.03)	6.56 (0.03)	6.59 (0.09)
	pH_45_SM	0.12	6.60 (0.03)	6.51 (0.03)	6.55 (0.09)
	ECULT	0.11	2.98 (0.10)	3.27 (0.12)	3.56 (0.34)
	ECUSM	0.20	3.76 (0.17)	4.27 (0.19)	4.13 (0.56)
	L* Minolta	**0.08**	45.6 (0.47)	47.3 (0.55)	47.9 (1.55)
	b* Minolta	**0.02**	3.26 (0.21)	3.99 (0.25)	5.05 (0.70)

SNPs g.-995A>G, .-311A>G and I199V formed the haplotype groups.

*P*-values significantly different at the nominal level are in bold.

*IMF* Intramuscular Fat, LT *M. Longissimus thoracis et lumborum* muscle, SM *M. semimembranosus muscle, n* number of genotypes.

**Table 7:** **Estimated least square means for significant meat quality traits in relation to copy number of the most abundant porcine *PRKAG3 *gene haplotypes as inferred by Arlequin for the promoter SNPs g.-995A>G and SNPg.-311A>G and non-synonymous SNP *I199V *locus in the Duroc breed (n = 98)**

**HAP ID**	**Traits**	***P*- value**	**Estimated trait mean per haplotype copy**		
			**0**	**1**	**2**
HAP1 (AAI) Freq: 0.19	IMFL%	0.15	1.72 (0.08)	2.01 (0.12)	1.88 (0.32)
	IMFS %	0.87	2.12 (0.09)	2.22 (0.14)	2.15 (0.39)
	Driploss%	**0.04**	2.61 (0.13)	2.30 (0.20)	1.24 (0.53)
	pHULT	0.13	5.63 (0.01)	5.67 (0.02)	5.67 (0.05)
	pHUSM	**0.02**	5.53 (0.01)	5.54 (0.02)	5.66 (0.04)
	pH_45_LT	0.52	6.57 (0.03)	6.57 (0.04)	6.44 (0.11)
	pH_45_SM	0.66	6.49 (0.03)	6.53 (0.04)	6.58 (0.11)
	ECULT	0.06	3.13 (0.10)	3.53 (0.16)	2.62 (0.44)
	ECUSM	0.35	3.99 (0.09)	3.77 (0.13)	4.14 (0.36)
	L* Minolta	0.32	46.4 (0.36)	45.8 (0.55)	44.3 (1.49)
	b* Minolta	0.09	3.73 (0.17)	3.04 (0.25)	2.96 (0.69)
HAP2 (AAV) Freq: 0.28	IMFL%	0.37	1.90 (0.09)	1.76 (0.08)	1.54 (0.30)
	IMFS %	0.99	2.17 (0.11)	2.15 (0.10)	2.11 (0.37)
	Driploss%	0.62	2.38 (0.15)	2.56 (0.16)	2.16 (0.53)
	pHULT	0.51	5.65 (0.01)	5.63 (0.01)	5.68 (0.05)
	pHUSM	0.59	5.54 (0.01)	5.53 (0.01)	5.52 (0.04)
	pH_45_LT	0.79	6.55 (0.03)	6.57 (0.03)	6.63 (0.11)
	pH_45_SM	0.08	6.52 (0.03)	6.48 (0.03)	6.71 (0.10)
	ECULT	0.93	3.21 (0.12)	3.26 (0.12)	3.34 (0.43)
	ECUSM	0.22	3.95 (0.11)	3.96 (0.11)	3.34 (0.34)
	L* Minolta	0.53	46.1 (0.42)	46.4 (0.41)	45.1 (1.43)
	b* Minolta	0.49	3.32 (0.21)	3.65 (0.19)	3.28 (0.68)
HAP3 (GGV) Freq: 0.28	IMFL%	0.49	1.86 (0.11)	1.81 (0.08)	1.51 (0.27)
	IMFS %	0.89	2.17 (0.12)	2.15 (0.10)	2.00 (0.33)
	Driploss%	0.24	2.39 (0.17)	2.41 (0.15)	3.25 (0.47)
	pHULT	0.21	5.65 (0.01)	5.64 (0.01)	5.57 (0.04)
	pHUSM	0.69	5.54 (0.01)	5.53 (0.01)	5.51 (0.04)
	pH_45_LT	0.89	6.56 (0.03)	6.57 (0.03)	6.52 (0.09)
	pH_45_SM	0.82	6.51 (0.03)	6.52 (0.03)	6.46 (0.11)
	ECULT	0.90	3.25 (0.13)	3.25 (0.12)	3.07 (0.38)
	ECUSM	0.44	4.04 (0.11)	3.84 (0.11)	3.93 (0.31)
	L* Minolta	0.99	46.1(0.45)	46.2 (0.40)	46.1 (1.29)
	b* Minolta	0.57	3.45 (0.21)	3.46 (0.19)	4.11 (0.60)
HAP4 (GAV) Freq: 0.21	IMFL%	0.06	1.76 (0.08)	1.98 (0.10)	1.28 (0.33)
	IMFS %	0.43	2.13 (0.11)	2.25 (0.13)	1.75 (0.41)
	Driploss%	0.38	2.35 (0.14)	2.67 (0.18)	2.25 (0.59)
	pHULT	0.56	5.65 (0.01)	5.63 (0.02)	5.64 (0.05)
	pHUSM	0.87	5.54 (0.01)	5.53 (0.01)	5.52 (0.05)
	pH_45_LT	0.95	6.56 (0.03)	6.57 (0.04)	6.55 (0.12)
	pH_45_SM	0.81	6.52 (0.03)	6.49 (0.04)	6.51 (0.12)
	ECULT	0.52	3.31 (0.11)	3.10 (0.15)	3.41 (0.47)
	ECUSM	0.11	3.80 (0.09)	4.07 (0.12)	4.49 (0.38)
	L* Minolta	0.37	45.8 (0.37)	46.8 (0.49)	47.2 (1.58)
	b* Minolta	0.57	3.38 (0.18)	3.69 (0.23)	3.29 (0.75)

SNPs g.-995A>G, .-311A>G and *I199V* formed the haplotype groups.

*P*-values significantly different at the nominal level are in bold.

*IMF* Intramuscular Fat, LT *M. Longissimus thoracis et lumborum* muscle, SM *M. semimembranosus muscle, n* number of genotypes.

HAP1 was associated with IMF% in the LTL muscle and driploss% in the Large White breed. In the Duroc breed, HAP1 was associated with driploss% and pHUSM. In both breeds, two copies of this haplotype had the most favourable effects on the associated phenotypes.

HAP2 was associated with IMF% in the LTL muscle, pHULT, pH_45_LT and pH_45_SM in the Large White breed. The presence of two copies of this haplotype had the most favourable effect on these associated traits. No associations were observed for HAP2 in the Duroc breed.

HAP3 was associated with driploss%, pHULT, pH_45_LT and b*Minolta in the Large White breed. The presence of two copies of this haplotype had the least favourable effect on these traits. No associations were observed for HAP3 in the Duroc breed.

HAP4 was present at too low a frequency (Freq: 0.01) in the Large White breed for subsequent analysis. No associations were observed for HAP4 in the Duroc breed.

### In-silico analysis of SNPs in relation to transcription factor binding sites in the promoter region

*In-silico* analysis of the SNP loci using five transcription factor binding site (TFBS) prediction tools (TFSEARCH, TESS, MatInspector, Match™ and ALIBABA2) is presented in Table 8. A CCAAT/enhancer binding protein site, encompassing the g.-995A>G site, was altered to a GATA binding site on substitution to the minor allele. This change was identified using all five tools.

**Table 8:** ***In-silico *analysis of transcription factor binding site motifs at SNP sites in the *PRKAG3 *promoter region**

**SNPs**	**Putative transcription factors**	**Predictor**	**Recognition sequence**	**Created with**	**Start**	**End**
g.-995A>G	**CCAAT/enhancer-binding protein (α and ß)**	TFSEARCH	CCCTTAGGC**A**ATAT _a_	A	−1004	−991
		TESS	CTTAGGC**A**ATAT			
		MatInspector	CCCCTTAGGC**A**ATAT			−991
		Match™ ALIBABA2	CCCCTTAGGC**A**ATATAGG			−991
					−1002	−989
					−1005	
	**HNF-4**	Match™	CCTTAGGC**A**ATA AA _c_			−992
						−992
			CCTGCCCCTTAGGC**A**AT		−1005	
					−100	−1010
g.-480C>T	**Pax-4**	Match™	C**C**GGGACCACCCACGAACTCC _b, c_	C	−481	−460
g.-461C>T	**SP1**	TFSEARCH	GCTGGGGAGGC**G**GAG _a b_	C	−472	−458
		TESS	C**G**GAGT GGGGAGGC**G**GA		−462	−457
		ALIBABA2			−469	−459
g.-311A>G	**IK-1**	Match™ TFSEARCH	CGGT**G**GGAACACA _a, c_	G	−315	−303
g.-221G>A	**Myoblast Determining Factors**	MatInspector	**C**GAGGACAGGTGAGAAG _b, c_	G	−221	−205
g.-30C>T	**RFX1**	Match™	CTGTATCTGGGCA**A**CAC _b, c_	T	−33	−17

SNP positions in recognition sequence are underlined. No transcription factor motifs were found in positions g.-968G>A, g.-671A>G, g.-583C>T, g.-158C>G.

^a^ Transcription factor binding site prediction changes on substitution to alternate allele.

^b^ Minus strand.

^c^ Muscle specific.

### Promoter assay

**Figure 5:**
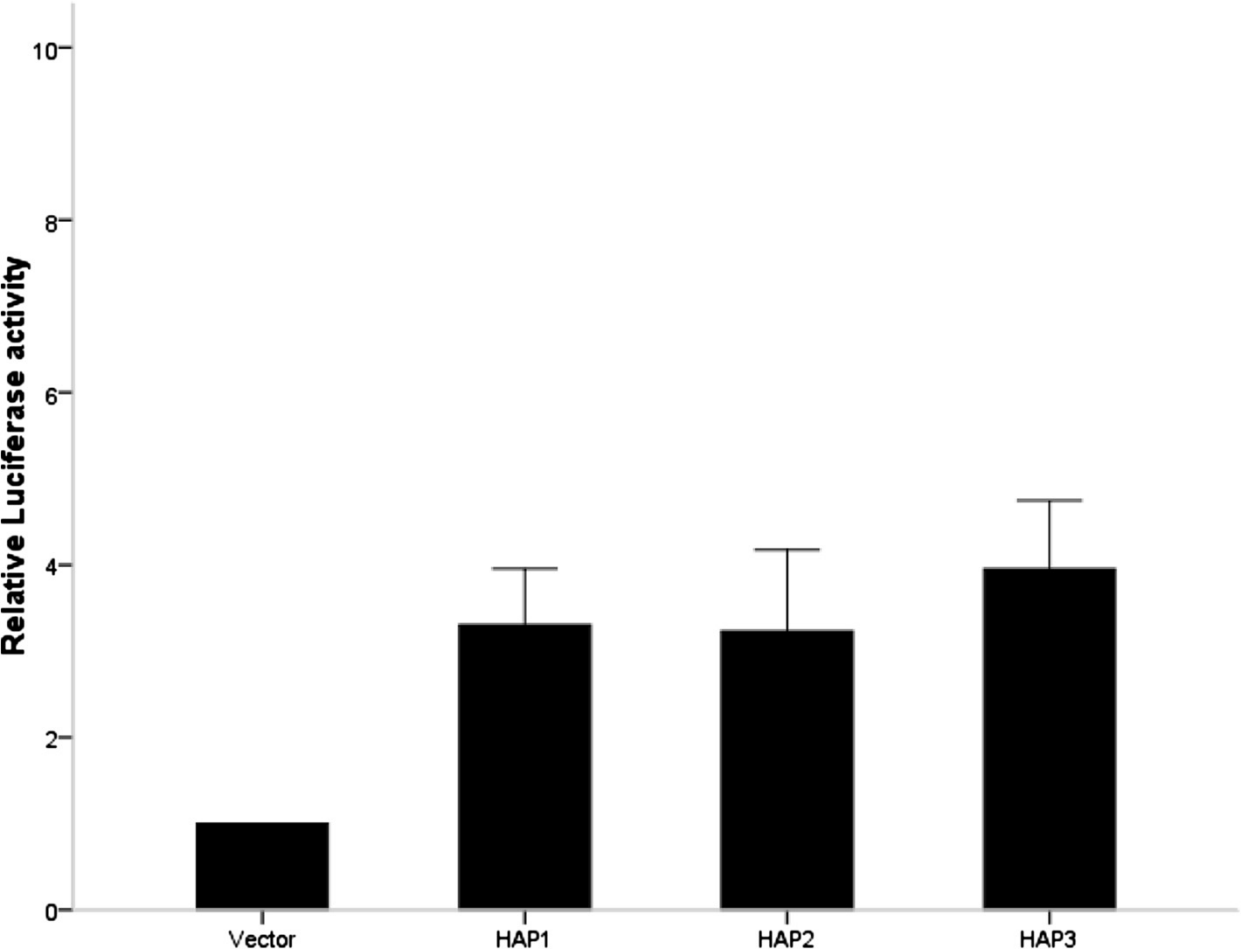
**The firefly luciferase values were normalised for total protein, and data is presented relative to the empty vector control assigned a value of 1.** The results are expressed as mean and the SE of three separate experiments performed in quadruplicate. Statistical analysis was performed using one way ANOVA.

The three major promoter haplotypes outlined in (Table 3) used in the *in vitro* promoter assay accounted for approximately 90% of the variation within the three breeds. Although no significant difference (*P* = 0.45) was observed between the haplotypes, transcriptional activity was established in the region cloned when tested *in vitro* in pre-adipocytes (Figure 5).

## Discussion

In this study, *PRKAG3* gene expression levels in porcine LTL muscle were correlated with a number of meat quality phenotypes including GP-influenced traits and IMF%. As SNPs in the regulatory region of a gene can influence the transcription rate [33], we explored the hypothesis that SNPs/inferred haplotypes in the *PRKAG3* promoter would be associated with pork quality. This hypothesis was further validated by identifying an association between g.-311A>G and *PRKAG3* gene expression and by the significant associations identified between g.-995A>G, g.-311A>G and quality traits pHULT, pH_45_LT, ECULT, driploss% and Minolta L* in the Large White pure bred population. The associations with non-synonymous SNP *I199V* in this study served to validate the studied phenotypic model and provided a useful comparator with which to view the novel promoter SNP associations. Inclusion of this much studied SNP also indicated that the study had sufficient statistical power to detect the influence of polymorphisms of moderate effect size. Ciobanu and colleagues highlighted the complexity of the phenotype/genotype relationship in the *PRKAG3* gene, proposing the model of “one gene-several polymorphisms-diverse phenotypes” when describing the phenotypic effects of the non-synonymous SNPs identified in the *PRKAG3* gene [7]. Our data supports this model as the novel promoter SNPs were not in significant linkage disequilibrium with the *I199V* locus, and exert different effects on phenotype which are independent from each other. This adds to the complexity of the phenotype/genotype relationship, and indicates that the additive effects of all these functionally distinct loci need to be considered in future studies on pork quality.

In this study, the associations which were observed between the *I199V* locus and a number of GP-influenced traits were in concordance with the wider literature in the breeds studied [7,13,17,19,22]. The association between *I199V* and IMF%, which was observed in both Large White and Pietrain breeds, is noteworthy because there is a lack of consensus in the wider literature on the relationship between *I199V* and IMF%. The association identified in our study, i.e. increasing IMF% from the VV to II genotype, is in concordance with two studies that report the same trend in a Korean native breed cross [26] and a Meishan cross [25]. The opposite trend was previously reported for a Landrace population [25] while several studies have reported a lack of association between *I199V* and IMF% [17,20,34]. Given the central role that *AMPK* has on all forms of energy metabolism, it is not surprising that variation that affects the function of its regulatory component might impact on lipid deposition in muscle. Associations between this gene region and lipid metabolism in muscle appear to be evolutionarily conserved as *AMPK/PRKAG3* is implicated as a molecular target to promote marbling in cattle [35] and triglyceride accumulation in mice [9].

The associations between *PRKAG3* gene expression and both GP-influenced traits and IMF% suggests that not only are protein structural modifications within the *PRKAG3* coding region influencing the meat quality traits studied, but that variability in the abundance of the transcript is also influential. This may be mediated by genetic variation in promoter activity since SNP g.-311A>G was significantly associated with gene expression. This hypothesis is further supported by the associations observed between both g.-995A>G and g.-311A>G and GP-influenced traits i.e. pHULT, driploss% and colour. Generally, the A allele in both promoter SNPs was associated both with improved meat quality based on glycolytic traits and reduced *PRKAG3* gene expression. HAP 2 and HAP 3 both have the same genotype V at the I199V locus but differ with respect to their effects on Driploss%, further supporting the effects of the promoter SNPs on phenotype. Drip loss is a significant challenge for the industry [36,37] and these haplotypes are potentially relevant to selection strategies aimed at improving this trait. Further to this, the promoter SNPs had highly significant associations with pHULT (*p* = 0.0003) in the Large White breed, an association which was not observed with *I199V*. Interestingly in this study, the associations with GP-influenced phenotypes are wider ranging and statistically more significant for the promoter SNPs than for *I199V* in the Large White breed. However, the associations with GP-influenced traits are more consistent across breeds for the *I199V* SNP and of slightly larger magnitude with respect to driploss%. It is noteworthy that in a recent study, a mouse model developed to over express *AMPK*γ reported a different phenotype to that expressing the gain of function variant of *AMPK*γ with respect to mitochondrial biogenesis indicating that the phenotypic effects of transcript abundance and mutations affecting protein function in *PRKAG3* are not equivalent in skeletal muscle [38].

One of the modes by which SNPs in the regulatory region of a gene can influence transcription rate is by forming or abolishing a TFBS [39]. In this study, the in-silico analysis suggested that g.-995A>G alters the associated transcription factor-binding site from a putative CCAAT/enhancer binding protein site to a putative GATA site on substitution to the minor allele. Both CCAAT/enhancer binding protein and GATA transcription factors have been implicated as key regulators of adipocyte differentiation and lipid metabolism [40], suggesting a potential role of this SNP in variation in IMF%. While g-311A>G was not associated with a putative TFBS site, it is positioned in a highly conserved 5' UTR, that contains several regulatory motifs characteristic of a core promoter [41]. 5' UTR regions have regulatory significance for translational efficiency and subsequent phenotype [42]. The substitution of SNP at g.-311A>G may affect the core promoter and thus modulate gene expression. This region also forms part of a 5' exon in the porcine transcriptome, referred to as ‘exon zero’ [41]. Exon zero is contained in one of the two known alternative transcripts of the *PRKAG3* gene. Hence based on *in silico* evidence it is possible that both these SNPs could have functional roles in the regulation of *PRKAG3*, however associations with *PRKAG3* gene expression were only observed for SNP g-311A>G and not g.-995A>G.

Taken together, the associations between *PRKAG3* promoter haplotypes, gene expression and phenotype gene suggest the possibility of differential promoter activity. While the promoter assay confirmed promoter activity in the region cloned in all the major haplotypes, the assay was unable to discern significant differences in transcriptional activity between the haplotypes in pre-adipocyte cells. It is however worth re-iterating that the transcription of *PRKAG3* is mainly ascribed to white skeletal muscle in humans and other species [5]. Furthermore the phenotypic associations observed were highly breed and tissue specific indicating both a highly specific and finely tuned transcriptional apparatus. It is therefore likely that changes in transcription rate mediated by variation in the transcription binding sites may only be realised *in vivo* as evidenced by the association between genotype at the g-311A>G locus and *PRKAG3* gene expression in the Large White cross.

In common with other recent porcine SNP association studies [29,43,44], the effects of putative functional SNPs can be breed and muscle specific. The effect of these SNPs was certainly not consistent across breed and muscle type studied. Breed seems to have important consequences for the phenotypic outcome of variation in both the regulatory and coding regions of this gene, and this was particularly evident for the promoter region. Alleles characterising HAP2 and HAP3 were associated with GP-influenced traits in the Large White breed only. Most of the associations observed in this study were seen in the LTL muscle. The relationship between muscle biochemistry and meat quality phenotypes has been shown to differ for the LTL and SM muscle [45]. *PRKAG3* gene expression is greater in LTL muscle [46] which displays more glycolytic traits compared to SM muscle [45]. In exercised trained pigs, carriers of the R200Q mutation has been shown to influence the relative fibre composition to varying degrees among functionally different muscles, thereby promoting a more oxidative phenotype [47]. With respect to the breed differences, the Large White has the largest percentage of fast glycolytic fibres in the LT muscle relative to the Pietrain and the Duroc breeds [45]. Hence, breed and muscle fibre type likely play interactive roles in defining the penetrance of the novel alleles in the *PRKAG3* promoter in relation to meat quality phenotypes.

While the overall goal of SNP/meat quality association studies is to provide markers to improve quality parameters in breeding populations, it is becoming increasingly apparent that there is significant breed variation in the potential for genetic improvement based on fixation of particular haplotypes in certain breeds. It is of interest that the *I199V* substitution effect on GP-influenced traits was conserved across all breeds, despite their divergent muscle characteristics. In contrast the promoter allele effect was apparent in the Large White breed, but not in the Duroc breed. This was also the case for the associations between gene expression and phenotype. It is noteworthy that the Duroc breed had superior meat quality characteristics as evidenced in earlier work [45,48]. This is supported by previous studies, where substantial genetic variation has been observed in *IGF2* gene and the *ANK1* gene regions in the Large White breed, but dramatically less in the Pietrain and Duroc breeds [29,43]. While Duroc and Pietrain are terminal breeds, in which carcass characteristics are the main selection criteria, the Large White breed is a maternal breed and therefore may retain a higher degree of genotypic and phenotypic diversity for meat quality traits. It is important to note however that there may have been a degree of ascertainment bias in SNP discovery in this study, because the genetic contribution of the Large White breed was approximately 33% in the Pietrain and Duroc cross-bred populations which were used for SNP discovery.

## Conclusions

To conclude, variation in the promoter region of the porcine *PRKAG3* gene has associations with meat quality phenotypes, including traits which are influenced by glycolytic potential and muscle metabolism in a breed-dependent manner. The novel SNPs presented here, combined with the *I199V* SNP, represent a new opportunity to select for reduced drip loss in terminal Large White sires based on combined novel genetic variation in the promoter, and known coding SNP in the *PRKAG3* gene. The lack of linkage disequilibrium observed between the promoter SNPs and the *I199V* locus infer that their effects on phenotype are independent hence the additive effects of all these loci should be considered in future studies on pork quality.

## Methods

### Animal resources

*M. longissimus thoracis et lumborum* (LTL) and *M. semimembranosus* (SM) tissues and blood were collected from two animal resources (86 crossbred and 295 purebred pigs) in which meat quality phenotypic data was available for a number of traits as previously described [43,45,46,49]. The cross-bred resource comprised of 31 Large White-sired, 23 Duroc-sired and 32 Pietrain-sired F1 female offspring with a common Large White × Landrace background. The purebred animals were females sampled from each of three closed populations (breeding lines) based on Large White (n = 98), Duroc (n = 99) and Pietrain (n = 98) and all reared in the same production system The purebred animals were reared in the same conditions and fed the same diet and were slaughtered at 140 days with a live weight of 109.56 ± 7.81 kg. [45]. The Pietrain line was homozygous dominant, NN (“Normal”) for the *RYR1 *[45,46].

### *PRKAG3* gene expression quantification

*PRKAG3* gene expression was quantified in the LTL tissue samples which were collected from the crossbred F1 animal resource for transcriptomic analysis. All tissue was taken from approximately the same posterior location, in RNAse free conditions and preserved in RNALater® (Ambion Ltd., Cambridge, UK) within 10 min post-exsanguination, snap-frozen in dry ice, kept overnight at 4°C, and then stored at −20°C. RNA was extracted using the Qiagen RNeasy® Fibrous Tissue Mini Kit (Qiagen, Hilden, Germany Ltd, West Sussex, UK) according to the manufacturer’s instructions, together with a DNase treatment. RNA integrity was assessed using the Bioanalyser 2100 RNA nano chip (Agilent Technologies, Santa Clara, California, USA) and quantified with the NanoDrop 1000 Spectrophotometer (Thermo Scientific, Waltham, MA).

Reverse transcription was carried out using total RNA to generate a cDNA template for use with the QuantiTect SYBR Green PCR Kit (Qiagen, Hilden, Germany). For the reverse transcription 2.5 μg of total RNA, 1 μl oligo (dT)12-18 (500 μg/ml), 1 μl of a 10 mM dNTP mix were combined together in a final volume of 12 μl, heated at 65°C for 5 min and then placed immediately on ice. The contents were collected by a brief centrifugation before adding 4 μl 5X first strand buffer, 2 μl 0.1 M DTT, 1 μl SUPERase-In (Ambion, Foster City) and 1 μl of Superscript III RNase H reverse transcriptase (200 u/μl) (Invitrogen, Carlsbad, CA). The reverse transcription was carried out at 50°C for 1 h followed by an enzyme inactivation step of 70°C for 15 min. The cDNA was diluted to 10 ng/μl for use as a template for quantitative PCR (qPCR).

All qPCR was performed on the Mx3000P™ Real-Time PCR System (Stratagene, La Jolla, California, USA) using the QuantiTect™ SYBR Green Kit (Qiagen, Hilden, Germany). qPCR reactions were carried out using 2X QuantiTect SYBR Green PCR Master mix, 1 μl of 10 mM sense and anti-sense gene specific primers, (final conc. 0.5 mM), 3 μl dH_2_O and 5 μl of total cDNA template (10 ng/ml) in a total volume of 20 μl. The thermal profile was 95°C for 15 min, followed by 40 cycles of 94°C for 15 s, 60°C for 30 s and 72°C for 30 s.

*PRKAG3* mRNA expression levels were determined for both splice variants using gene specific primers [Genbank:NM_2140777]:

Forward: 5' CTCCGACTCCAACACAGACCATCT 3',

Reverse: 5' TTCTGCAGCTCATCATCCCAGC 3'. *PRKAG3* gene expression was normalised using the geometric mean of reference genes: Ribosomal protein L4 (*RPL4):* [Genbank: DQ845176]:

Forward: 5' AGAGATCCAAAGAGCCCTCCGC 3' and

Reverse 5' GCCTGGCGAAGAATGGTGTTTC 3' and TATA box binding protein (*TBP)*: [Genbank: DQ845178]: Forward: 5' TTAATGGTGGTGTTGTGGACGGC 3', Reverse: 5'CCAAATAGCAGCACAGTACGAGCAA 3'. These reference genes were previously found to be stable for gene expression analysis in LTL muscle [50]. Stability was re-confirmed on these samples using Genorm [51]. PCR efficiencies (E) was calculated for each target *PRKAG3* (99%), *RPL4* (102%) and *TBP* (99.9%) and hence were suitable for comparison. The relative expression was calculated according to an established protocol [52,53].

### DNA preparation, promoter SNP discovery and SNP genotyping

Genomic DNA was extracted from LTL muscle tissue samples using DNeasy kit from Qiagen (Qiagen, Hilden, Germany) or from whole blood using the Wizard Genomic DNA Purification Kit (Promega, Madison, USA). DNA quantity and purity (A260/A280 ratio) for each sample was assessed using the NanoDrop™ 1000 Spectrometer (Thermo Scientific, Waltam, MA, USA).

The promoter region of the *PRKAG3* gene (1322 bp long) was sequenced in the crossbred F1 population.

Primers were designed from draft sequence of a BAC clone (GenBank Ref. AY263454) which contained sequence flanking the *PRKAG3* gene [41]. Primers were designed using the web based application Primer 3 [54].

A 1322 bp fragment located in the promoter region was amplified using 20 pmol of primer: Forward: 5′ AGGGATGCTGCAGAAGAAGA '3 and Reverse: 5′ CACACAGAACCGCACAGACT '3, 20 ng genomic DNA using Qiagen PCR Master Mix (Qiagen, Hilden, Germany) in a 50 μl volume. The PCR conditions for the touchdown PCR reaction are as follows: 95°C for 2 min, and for 14 cycles; 95°C for 30 s, 62.3°C (decrease 0.5°C per cycle) for 30 s, 72°C for 2 min 20 s. Followed by 19 cycles; 95°C for 30 s, 53.3°C for 30 s and 72°C for 2 min, 20 s.

PCR products were analysed by agarose gel electrophoresis (2%) and ethidium bromide staining and visualised on MultiDoc Imaging System (UVP, Upland, CA, USA). Products were purified prior to sequencing using GenElute™ Mammalian Genomic DNA Miniprep Kit (Sigma-Aldrich Corp., St. Louis, MO, USA) and quantified on a NanoDrop™ 1000 Spectrometer (Thermo Scientific, Waltam, MA, USA). Sequencing of the purified PCR product was carried out in both directions by Eurofins MWG-Biotech (Ebersberg, Germany). Sequences were aligned and data analysed using MEGA® (Molecular Evolutionary Genetic Analysis) v 4.0 software [55]. The *I199V* locus was genotyped using nested PCR followed by restriction digest with 10 U of Hga1 (Fermentas, Vilnius, Lithuania) at 37°C for 6 hrs [18].

### Tagging SNP analysis

Based on patterns of linkage disequilibrium in crossbred animals, minor allele frequencies and the ability to characterise haplotype blocks, two SNPs (g.-995A>G, g. SNP -311A>G) in the promoter region and one in the coding region *I199V* were selected for association analysis in the three purebred populations. Genotyping of SNPs g.-311A>G and *I199V* was performed using the Sequenom iPLEX assay (Sequenom, Hamburg, Germany). SNP g.-995A>G was genotyped using a custom TaqMan assay, Assay ID: AHN1HPD (Applied Biosystems, Warrington, UK).

### Phenotypic analysis

Meat quality phenotypic information for LTL and SM were available for cross-bred samples [49] and purebred samples [46] as previously described. pH and temperature at 45 min and 24 hr were measured in cross-bred samples as detailed in [49] and in pure-bred animals according to [46]. For both sets of LTL samples, drip loss (driploss %) was determined after 3 days according to the method of Honikel (1998) and expressed as a percentage of the initial weight [56]. Percentage cooking loss (cookloss %) was measured in cross-bred samples only and was determined by weighing transverse sections of the LTL before and after they were heated to a core temperature of 75°C in a circulating water bath held at 77°C. Electrical conductivity for both sets of samples was measured at a frequency of 1 KHz using a Pork Quality Meter (PQM, INTEK, Aichach, Germany) in accordance with manufacturer’s instructions. Bloomed CIE L* (lightness), a* (redness) and b* (yellowness) values were determined in the LTL at 7 days post mortem in the cross-bred animals as detailed in [49] and for pure-bred pigs at the last rib at 1 day post mortem using a Minolta C2002 Spectrophotometer (Minolta, Japan) as described in [46]. For cross-bred animals, intramuscular fat (IMF%) concentrations were determined in thawed minced LTL samples using the Smart System 5 microwave moisture drying oven and NMR Smart Trac Rapid Fat Analyser (CEM Corporation USA) using AOAC Official Methods 985.14 & 985.26, 1990. In purebred samples, IMF% levels in LTL and SM were assessed using a Near Infrared Spectroscopy apparatus [46].

### Promoter assay

The primers used to amplify the promoter region were modified (modification highlighted in bold) at the 5' end to contain HIND III and BGL II restriction sites in the Forward:

5' CCTTAGATCTGGGATGCTGCAGAAGAAGAG 3’ and Reverse: 5' GGATAAGCTTAGGAGTGCGCAACACTGTATC 3’ primers, respectively.

The promoter region was amplified using 80 ng DNA, 45 μl Platinum High Fidelity Master Mix (Invitrogen Carlsbad, California, USA) and 20 ng primer in a final volume of 50 μl. The PCR conditions were as follows 95°C, 5 min, and for 30 cycles; 95°C for 1 min, 60°C for 1 min and 72°C for 2 min. This was followed by a single step of 72°C for 5 min. Samples were purified and quantified.

The PCR products for each haplotype (1 μg) and (4 μg) reporter vector pGL 4.17 (Promega, Corp., Madison, WI, USA) were then digested using HIND III (Promega, Corp., Madison, WI, USA) at 37°C for 3 hr and purified using GenElute PCR Purification Kit (Sigma-Aldrich Corp., St. Louis, MO, USA) and then digested with BGL II (Promega, Corp., Madison, WI, USA) at 37°C for 3 hr. 100 ng of vector and 70 ng of digested product were then ligated using T4 DNA Ligase (Promega, Corp., Madison, WI, USA) at 4°C.

Chemically competent *E. Coli* XL1 Blue host cells were transformed with the ligated product 5 μl and selected on LB medium containing ampicillin (100 μg/ml). The genotypes of the positive clones were verified with DNA sequencing (Eurofins MWG-Biotech, Ebersberg, Germany).

### Cell culture and transient transfection assay

Mouse 3T3-L1 pre-adipocytes were obtained from the American Type Culture Collection (ATCC, Manassas, VA, USA). Cells were cultured in a dulbecco modified eagle’s medium (DMEM, Gibco, Invitrogen Corp., San Diego, CA, USA) containing 10% fetal calf serum (Gibco, Invitrogen Corp., San Diego, CA, USA) and 1% penicillin-streptomycin (Sigma-Aldrich Corp., St. Louis, MO, USA) in a 37°C humidified incubator with 5% CO_2_. Medium was replaced every alternate day.

The day prior to transfection, 3T3-L1 cells were cultured 3 ×10^4^ per ml cells in DMEM containing 10% fetal calf serum in a 24 well cell culture plate (Greiner Bio-One, Gmbh, Germany) in a 37°C humidified incubator with 5% CO_2_. The transfection cocktail (for each well) contained 25 μl DMEM basal media, 0.8 μl FuGENE HD Transfection Reagent (Roche Diagnostics GmbH, Mannheim, Germany) and 200 ng of endotoxin free *PRKAG3* promoter construct. Following incubation at room temperature for 15 min, this cocktail was introduced drop wise onto the cells. Cells were then grown on a DMEM containing 10% fetal calf serum for 24 hr.

After 24 hr the media was removed and the cells washed with 500 μl of sterile phosphate buffer saline. Lysis of the cells was performed by adding 200 μl of passive lysis buffer (Promega Corp., Madison, WI, USA) followed by incubation at 37°C in a shaking incubator for 30 min at 800 rpm. Luciferase activity was measured in 20 μl of cell lysate after adding 100 μl luciferase assay reagent in a luminometer GLOMAX™, (Promega Corp., Madison, WI, USA). All measurements were normalised to the total protein.

### Identification of putative regulatory SNP’s in the *PRKAG3* promoter region

To identify SNPs that putatively affect promoter elements, the upstream sequence containing the SNPs was screened for the presence of putative selective transcription factor binding sites in silico using five prediction tools including;TFSEARCH [57], TESS [58], MatInspector [59], AliBaba2 [60] and MATCH [60].

Particular attention was paid to binding sites which changed on substitution to the minor allele as well as sites known to be of relevance to mammalian muscle with high similarity scores and matrix similarities.

### Statistical methods

#### Haplotype analysis

Estimation of SNP frequencies and tests for departure from Hardy Weinberg equilibrium were carried out using the Excel Microsatellite Toolkit [61] at each locus for each breed. Linkage disequilibrium and haplotype blocks were identified using Haploview [62]. Following haplotype inference the ELB algorithm was used to assign the most likely haplotype combination to each individual animal as implemented using Arlequin [32].

### Association analysis

The relationship between normalised expression of *PRKAG3* and meat quality traits was calculated using Pearson correlation in PASW Statistics 18.0 software (SPSS, Inc., Somers, NY, USA). Association analysis was carried between genotyped SNPs/haplotypes and values of meat and carcass quality traits in each breed using the least square means method of GLM (General Linear Model) procedure in SAS (version 9.1; SAS Institute, Cary, NC, USA). Slaughter date was included in the model as a covariate. Each analysis tested for a difference in least square means of meat and carcass quality traits where 0, 1, or 2 copies of each haplotype were present.

The significance of the *in vitro* reporter assay across the three haplotypes and the association between genotypes and *PRKAG3* expression were established using One-Way ANOVA, in PASW Statistics 18.0 software (SPSS, Inc., Somers, NY, USA).

## Abbreviations

AMPK: Adenosine monophosphate activated protein kinase; SNPs: Single nucleotide polymorphisms; GP: Glycolytic potential; IMF: Intramuscular fat; LTL: *M. longissmus thoracis et lumborum* muscle; SM: *M. semimembranosus* muscle; pHULT: pH at 24 hr in LTL; pHUSM: pH at 24 hr in SM; pH_45_LT: pH at 45 min in LTL; pH_45_SM: pH at 45 min in SM; ECULT: Electrical conductivity in LTL; ECUSM: Electrical conductivity in SM; MAF: Minor allele frequency; TFBS: Transcription factor binding sites; UTR: Un-translated region; qPCR: Quantitative polymerase chain reaction.

## Competing interests

The authors declare that they have no competing interests.

## Authors’ contributions

MR carried out laboratory work, collation of data analysis, bioinformatic and population data analysis and prepared the first draft of the manuscript. AH, CM and RMH carried out laboratory work relating to DNA purification, qPCR and TaqMan genotyping respectively. JMB and MG were involved in determination of meat phenotypes for gene expression study and association analysis respectively and OS contributed to determination of phenotypes and DNA extraction. TS, RMH, AMM and GD designed and coordinated the study. TS, RMH and MR edited the manuscript and participated in the development of the final draft. All authors agreed with the final manuscript.
